# Editorial: Improving Neuroprosthetics Through Novel Techniques for Processing Electrophysiological Human Brain Signals

**DOI:** 10.3389/fnins.2022.937801

**Published:** 2022-06-09

**Authors:** Marianna Semprini, Gabriele Arnulfo, Ioannis Delis, Felix Siebenhühner, Gianluca Susi

**Affiliations:** ^1^Rehab Technologies, Istituto Italiano di Tecnologia, Genova, Italy; ^2^Department of Informatics, Bioengineering, Robotics and System Engineering (DIBRIS), University of Genova, Genova, Italy; ^3^School of Biomedical Sciences, University of Leeds, Leeds, United Kingdom; ^4^Neuroscience Center, HiLife, Helsinki University, Helsinki, Finland; ^5^Department of Structure of Matter, Thermal Physics and Electronics, Complutense University of Madrid, Madrid, Spain; ^6^Center for Cognitive and Computational Neuroscience, Complutense University of Madrid, Madrid, Spain

**Keywords:** computational model, EEG, stereo-EEG (SEEG), artifact removal, BCI—brain computer interface

Neurological diseases causing motor and/or cognitive impairments are among the most common causes of disabilities in adults. Recent advancements in neurorehabilitation and neuroprosthetics have led to a growing use of brain-computer interfaces (BCIs) and neuromodulatory interventions in clinical settings to treat neurological diseases such as Parkinson, stroke, epilepsy, schizophrenia (Semprini et al., 2018). However, additional research is needed to maximize therapy efficacy. New research should focus on decoding the nervous system information flow and on understanding the neural mechanisms underlying increased plasticity manifesting after prolonged rehabilitation treatment or prosthetic use. The study of electrophysiological brain signals enables tackling these challenges on several levels (Figure 1). For example, computational models are proving to be a valuable tool both for the optimization of stimulation procedures (Merlet et al., 2013; Seo and Jun, 2017) and the understanding of reconfiguration mechanisms following prosthetic use (Collinger et al., 2013). Additionally, the identification of novel biomarkers would make it possible to progressively refine and intensify the employment of computational models, as well as to improve diagnostic frameworks and develop new personalized neuroprosthetic applications (Hayashibe et al., 2015; Ubeda et al., 2021). In order to fully exploit the potential of these novel techniques, more standardized analysis protocols of electrophysiological brain signals will be necessary.

**Figure 1:**
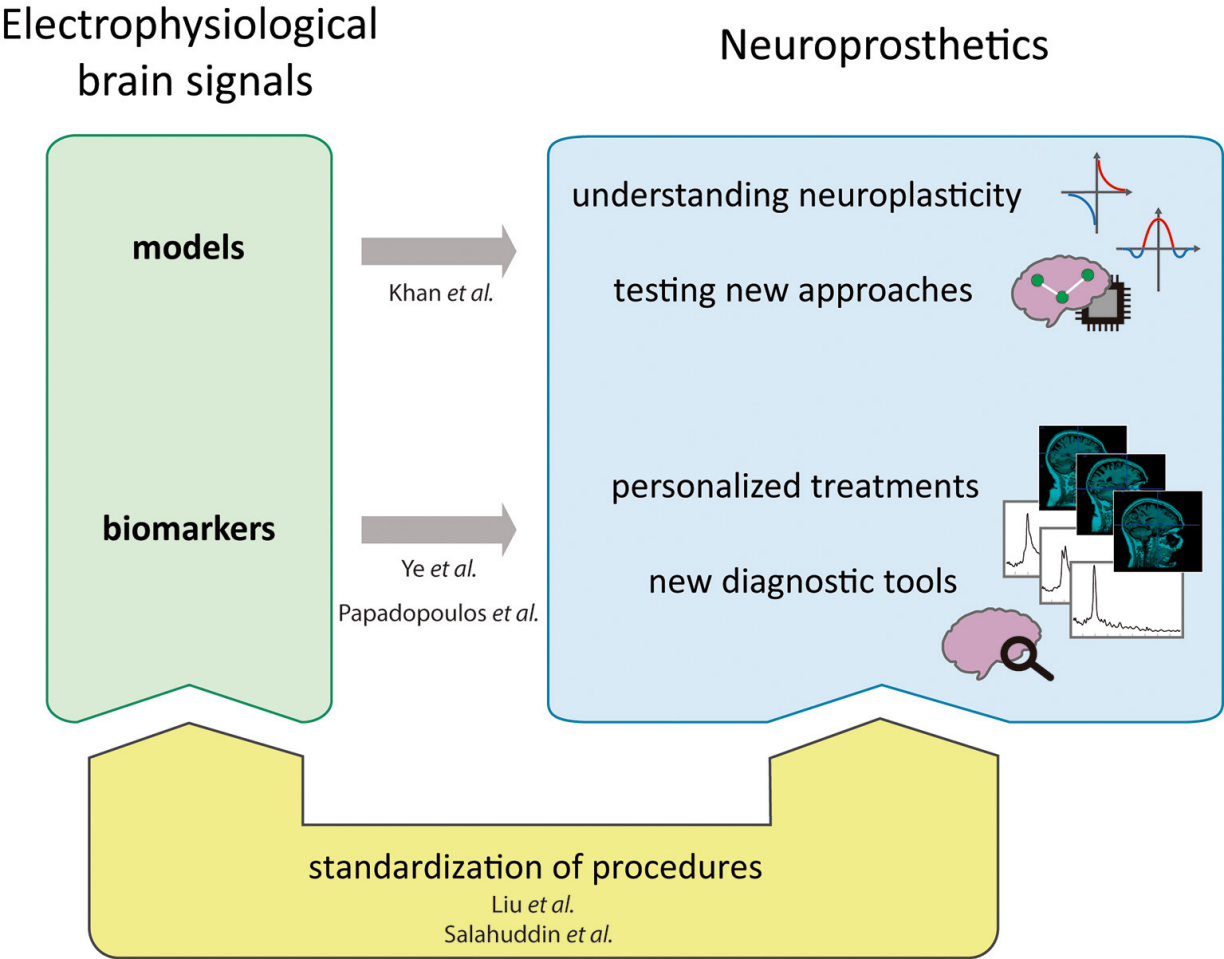
A framework for the adoption of non-invasive electrophysiological brain signals for neuroprosthetics and how the presented works fit in it.

Addressing the aforementioned issues will pave the way for innovative neurorehabilitation methods, leading to a fuller recovery of functions. This Research Topic thus focuses on innovative approaches for translating current research results into useful clinical applications and novel therapeutic possibilities in the neurorehabilitation and neuroprosthetic field. We collected a total of five studies centered on these topics: two studies focusing on electrophysiological signal acquisition and processing (Liu et al.; Salahuddin et al.); one proposing novel methods to extract useful features from stereo-electroencephalography (SEEG) (Ye et al.); one describing an open source dataset of healthy and pathological electroencephalographic (EEG) signals (Khan et al.); and one recommending the use of motor imagery BCIs for rehabilitation purposes (Papadopoulos et al.).

The article titled *Investigating Data Cleaning Methods to Improve Performance of Brain–Computer Interfaces Based on Stereo-Electroencephalography* by Liu et al. aims at comparing performance improvement in hand gestures decoding by adopting different data cleaning approaches. In this work, the authors further suggest the importance of appropriate referencing schemes in invasive recordings and their impact in SEEG-based BCI tasks.

Salahuddin et al. present a review titled *Signal Generation, Acquisition, and Processing in Brain Machine Interfaces: A Unified Review*, which aims to connect latest developments and relevant open questions in this field, embracing signal generation, acquisition, and processing stages. The authors focus on the developments of the last decade and discuss possible solutions to improve their presence in the market.

In the manuscript titled *Spontaneous State Detection Using Time-Frequency and Time-Domain Features Extracted From Stereo-Electroencephalography Traces*, Ye et al. investigate time- and frequency-based features for the decoding of intermitted auditory stimuli. This work brings additional evidence that a combination of multiple time and frequency features improve decoding beyond the classical event-related amplitude modulations.

Khan et al. present *The NMT Scalp EEG Dataset: An Open-Source Annotated Dataset of Healthy and Pathological EEG Recordings for Predictive Modeling*. In this article they introduce a large novel labeled EEG dataset and use several published machine learning (ML) models to discriminate abnormal from normal EEGs. This work not only provides a novel, robust EEG dataset, freely available to the scientific community, but also highlights how a diverse and heterogeneous dataset is a key asset when training ML models, and how new datasets are needed to progress the field. Importantly, the authors introduce a tool for automated signal screening, which is a fundamental prerequisite for real-time neuroprosthetic applications.

Papadopoulos et al. propose *An Impending Paradigm Shift in Motor Imagery Based Brain-Computer Interfaces*. In this manuscript, the authors offer a perspective on novel methods for interfacing with the nervous system in the neurorehabilitation context and advocate for refined neurophysiological markers stemming from subject-specific features extraction to improve non-invasive BCIs. This work adds to the growing body of literature promoting the development of novel biomarkers and of personalized approaches.

The articles collected in this Research Topic illustrate how non-invasive electrophysiological signals can be exploited for future neurorehabilitative applications (Figure 1). Indeed, new datasets such as the one proposed by Khan et al. are needed to test computational models describing new therapeutic approaches and neuroplasticity mechanisms underlying recovery. Moreover, the identification of novel biomarkers based on brain electrophysiological recordings will enable new diagnostic tools and personalized approaches, as proposed by Ye et al. and Papadopoulos et al. Finally, standardization of signal processing techniques as suggested by the work of Liu et al. and Salahuddin et al. is necessary to ensure the adoption of electrophysiological brain signals for clinical applications based on neuroprosthetics. Overall, the development of these novel solutions could help reduce the obstacles faced by people with disabilities and improve their quality of life.

## Author Contributions

MS, GA, and GS outlined the manuscript. MS wrote the first draft of the manuscript. GS prepared the figure. All authors contributed to the writing and approved the final version.

## Conflict of Interest

The authors declare that the research was conducted in the absence of any commercial or financial relationships that could be construed as a potential conflict of interest.

## Publisher's Note

All claims expressed in this article are solely those of the authors and do not necessarily represent those of their affiliated organizations, or those of the publisher, the editors and the reviewers. Any product that may be evaluated in this article, or claim that may be made by its manufacturer, is not guaranteed or endorsed by the publisher.
